# Innate immune modulation in transplantation: mechanisms, challenges, and opportunities

**DOI:** 10.3389/frtra.2023.1277669

**Published:** 2023-12-08

**Authors:** Corinne E. Praska, Riccardo Tamburrini, Juan Sebastian Danobeitia

**Affiliations:** ^1^ Division of Transplantation, Department of Surgery, University of Wisconsin, Madison, WI, United States; ^2^ Baylor Annette C. and Harold C. Simmons Transplant Institute, Baylor University Medical Center, Dallas, TX, United States

**Keywords:** innate immune system, transplantation, complement, toll-like receptor, ischemia-reperfusion injury, therapeutics

## Abstract

Organ transplantation is characterized by a sequence of steps that involve operative trauma, organ preservation, and ischemia-reperfusion injury in the transplant recipient. During this process, the release of damage-associated molecular patterns (DAMPs) promotes the activation of innate immune cells via engagement of the toll-like receptor (TLR) system, the complement system, and coagulation cascade. Different classes of effector responses are then carried out by specialized populations of macrophages, dendritic cells, and T and B lymphocytes; these play a central role in the orchestration and regulation of the inflammatory response and modulation of the ensuing adaptive immune response to transplant allografts. Organ function and rejection of human allografts have traditionally been studied through the lens of adaptive immunity; however, an increasing body of work has provided a more comprehensive picture of the pivotal role of innate regulation of adaptive immune responses in transplant and the potential therapeutic implications. Herein we review literature that examines the repercussions of inflammatory injury to transplantable organs. We highlight novel concepts in the pathophysiology and mechanisms involved in innate control of adaptive immunity and rejection. Furthermore, we discuss existing evidence on novel therapies aimed at innate immunomodulation and how this could be harnessed in the transplant setting.

## Introduction

The immune system consists, broadly, of two intricately interconnected arms: the innate and the adaptive. The innate arm is responsible for a rapid and broad response to invading pathogens, as well as to diverse physical and metabolic insults (1). This response involves the activation of a complex network of cellular and soluble mediators of inflammation that play a crucial role in maintaining immune surveillance at critical tissue sites. However, the failure to resolve the response can lead to persistent inflammation, tissue damage, and fibrosis (2, 3). The innate immune system uses pattern recognition receptors (PRRs) to detect conserved pathogenic motifs, such as lipid and carbohydrate moieties. PRRs are expressed on various immune and non-immune cells and can sense pathogen-associated molecular patterns and danger signals. Toll-like receptors (TLRs) are the most extensively studied PRRs, found on the cell surface and endosomes of cells, and they recognize bacterial lipopolysaccharides and viral nucleic acids. Intracellular signaling pathways are initiated upon PRR activation, resulting in the mobilization of inflammatory and immune responses (4, 5). The innate immune system's hard-wired responsiveness provides a critical first line of defense against invading pathogens and tissue damage, ensuring a rapid and effective response to potential threats.

In contrast, the adaptive arm is triggered by the presence of specific antigens, and it involves a highly targeted and specific response. This response is characterized by clonal expansion and affinity maturation of T and B cells, resulting in the generation of high-affinity receptors for antigenic peptides (6). The innate arm plays a vital role in initiating and enhancing the adaptive response by providing signals that trigger the recruitment and activation of cells of the adaptive immune system. The coordinated and integrated interplay between the innate and adaptive arms is essential for the acquisition of effective and long-lasting immunity against invading pathogens (7). The innate arm provides a rapid and non-specific response, while the adaptive arm provides a highly specific and targeted response. Therefore, the ability of the immune system to generate a successful defense against invading pathogens depends on the functional interdependence between these two arms of the immune system (8).

The innate pathway has been extensively studied in the context of transplantation. Understanding the role of these innate pathways in graft dysfunction and rejection is of paramount importance. By exploring the intricate interplay between the innate immune system and the transplantation process, we can identify novel therapeutic targets for promoting graft acceptance.

This review aims to provide a comprehensive analysis of the complement and TLR systems in the context of transplantation. We will examine the mechanisms underlying their activation, their contribution to graft dysfunction and rejection, and the potential for targeted manipulation of specific components to steer the immune response towards graft protection and acceptance. In doing so, we hope to deepen our understanding of the complex immune interactions that underlie innate immune responses in transplantation and to provide valuable insights into the development of new immunomodulatory strategies for improving transplant outcomes.

### Components of the innate immune system and their role in transplant

#### Complement

**Figure 1:**
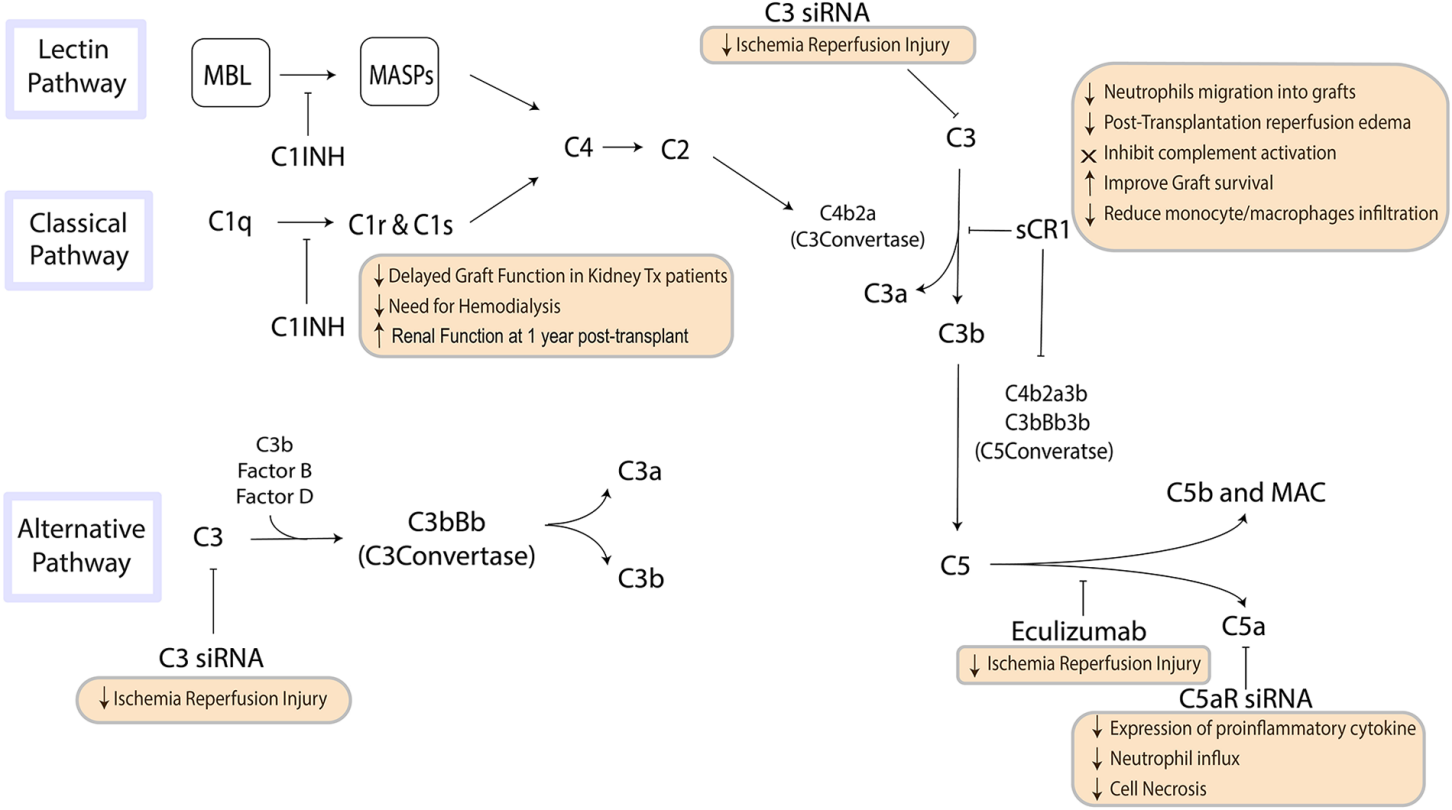
The pathways of the complement system are a crucial component of innate immunity that influence inflammation and the recruitment of the adaptive immune system. Steps in this pathway can be targeted to suppress inflammation and influence immune response to organ transplantation. MBL, mannose-binding lectin; MASP, mannan-binding lectin-associated serine protease; MAC, membrane attack complex.

The complement system is a vital component of the innate immune system. It is triggered by pattern recognition molecules (PRMs) that recognize specific patterns on the surface of invasive pathogens or damaged cells. The classical and lectin complement activation pathways converge on C3, the pivotal component of complement, while the alternative pathway generates a C3 convertase from C3b and factor B (fB). The resulting cleavage of C3 generates complement effectors, including the membrane attack complex (MAC), C3a, and C5a, which recruit the adaptive immune system and mediate inflammation (Figure 1) (9, 10).

Allograft rejection is a complex process that involves both innate and adaptive immune responses, and complement activation has been widely implicated in the initiation and progression of allograft injury during ischemia reperfusion injury (IRI) and in the pathophysiology of rejection (10–12). Complement activation during IRI is triggered by locally released CL-11, which activates C3a/C3aR interaction leading to glomerular and tubular injury and stimulates secondary epithelial cell chemokine production that contributes to local inflammation (13, 14).

The role of complement activation in acute rejection is more complex. The inflammatory response triggered by complement activation in a transplanted organ is mediated via generation of biologically active complement fragments. These fragments mediate chemotaxis and activate neutrophils and macrophages, resulting in a cascade of biological effects that depend on the site and trigger of complement activation (15). Once the MAC is formed, cells are induced to produce IL-1α and IL-8, further increasing local tissue inflammation (16).

Complement activation is detrimental in itself, as it damages the transplanted organ, but it also leads to recruitment of the adaptive immune system by enhancing T-cell activity. C5a has been shown to be a strong chemoattractant, and C5aR deficiency has demonstrated a protective effect in mouse models of IRI (17). Understanding the varying roles of complement within the transplant process is critical to predicting transplant outcomes and developing strategies to minimize the risk of allograft rejection.

#### Toll-like receptors

IRI is a multifaceted process that entails the activation of diverse pathways such as toll-like receptor (TLR) signaling, extracellular signaling molecules, alterations in gene expression, production of reactive oxygen species (ROS), regulation of cell death, activation of innate and adaptive immune factors, and the triggering of hypoxia-inducible factors (18). In general, this process constitutes a classic positive feedback loop in which damaged cells at the transplantation site release redox-sensitive damage-associated molecular patterns (DAMPs) that promote local accumulation of recipient-derived monocytes and polymorphonuclear leukocytes (PMNs), such as neutrophils (19). These cells sustain immune cascades and amplify the destruction of foreign tissue. This self-amplified cytotoxic cascade is initiated by the release of DNA/RNA complexes and/or acetylation of the non-histone chromatin-associated protein such as high mobility group box 1 (HMGB1), a representative danger signal. Additionally, degradation of the extracellular matrix can release molecules such as heparan sulfate, hyaluronan, fibrinogen, fibronectin A domain, or tenascin C, which can further amplify the response to tissue damage (20).

Acute kidney injury (AKI) is a complex disorder characterized by a rapid decline in renal function resulting from various etiologies. Neutrophils and macrophages are among the earliest effector cells involved in AKI, where they are found to persist for several days. The contribution of these cells to the pathophysiology of IRI results from their adherence to endothelium, release of ROS and proteases, and their ability to synthesize and secrete cytokines/chemokines that recruit other effector cells to the organ, thereby amplifying local inflammation (21–23).

Zhang et al. investigated the inactivation of neutrophils using mAbs against high mobility group box 1 (HMGB1) in a mouse model of AKI. They found that the treatment alleviated IRI-induced renal dysfunction by suppressing the activation of the HMGB1-Toll-like receptor 4 (TLR4)-interleukin (IL)-23-IL-17A signaling axis. This resulted in the reduction of neutrophil-mediated inflammation, chemokine expression, oxidative stress, and apoptosis (24). Neutrophil trafficking is also dependent on the crosstalk between donor non-classical and classical monocytes in a lung transplant IRI model (25).

Depleting neutrophils entirely may not be clinically feasible, given their critical role in pathogen clearance and immune surveillance. Instead, selectively blocking the recruitment of circulating leukocytes to the inflammatory site may be a better approach. In a recent study, researchers used mAb treatment to block the vascular surface marker CD321, a marker for the transmigration of circulating leukocytes into the inflamed tissue. This blockade attenuated damage responses by hepatic IRI, evaluated by serum liver enzymes, inflammatory cytokines, and hepatocyte cell death (26).

#### The IL-6 pathway

As a critical regulator of inflammation, IL-6 plays a pivotal role in the pathophysiology of transplant rejection (27). It is noteworthy that upregulation of IL-6 during brain death promotes a proinflammatory state even before organ procurement (28, 29), and during cold preservation, there is a detectable increase in graft IL-6 levels (30). This increase in IL-6 then upregulates adhesion molecules, inflammatory cytokines such as IL-17 and IFN-γ, and molecules that regulate migration in endothelial cells (31). Importantly, a growing body of evidence suggests that IL-6 blockade can mitigate the pro-inflammatory environment associated with brain death and IRI (32). Evidence from experimental and human studies links IL-6 to allograft injury, with blockade of the IL-6/IL-6R signaling pathway shown to mitigate these effects (33, 34). In mouse skin transplantation models, IL-6 promotes T cell alloreactivity and impairs Treg function (35). In addition, inhibition of IL-6 signaling reduces the production of donor-specific antibodies and modulates immune regulatory and effector cells (36, 37). Studies in murine models demonstrate that IL-6 plays a critical role in the pathogenesis of acute and chronic allograft rejection, and blocking IL-6 reduces/prevents rejection and fibrosis (38–41). In human transplant recipients, higher levels of IL-6 are consistently associated with worse outcomes, including a higher risk for acute rejection (42–44).

#### Coagulation cascade

The coagulation system, through the extrinsic and intrinsic pathways, activates factor X. Initiated by vascular injury, the extrinsic pathway catalyzes thrombin production, vital for stimulating platelets and vascular cells. The intrinsic pathway is autoactivated when circulating factor XII contacts negatively charged surfaces, under the regulation of antithrombin, tissue factor pathway inhibitor, and activated protein C. Importantly, coagulation interacts closely with the innate immune system and the complement cascade, influencing thrombogenesis and fibrinolysis (45).

Thrombin and tissue factor interact with protease-activated receptors on immune cells, releasing inflammatory mediators crucial for transplantation and IRI. In delayed graft function (DGF), ischemia/reperfusion-triggered coagulation activation, fibrin deposition, and robust expression of pro-inflammatory agents primes adaptive alloimmune responses and leads to allograft dysfunction (46, 47).

Mechanistically, this is explained by infiltrating dendritic cells expressing protease-activated receptor 1 (PAR-1), which influences cytokine gene expression and enhances T helper-1 bias in DGF (48). Interstitial coagulation activation, indicative of renal graft rejection, involves fibrin stimulation of fibrosis-contributing cells, and post-transplantation complications like microvascular thrombi, endothelial dysfunction, and fibrin deposition can lead to graft dysfunction, particularly in highly sensitized recipients (49, 50).

The fibrinolytic system, responsible for removing fibrin, prevents vessel occlusion and is significant in transplantation (51). The cause of organ donors' death can affect fibrinolysis activation, with injured donors exhibiting enhanced fibrinolytic activity (52). DGF correlates with increased urokinase (uPA) and urokinase-type plasminogen activator receptor (uPAR) expression, which impacts graft function and renal filtration. Chronic renal graft failure is characterized by fibrin deposition due to a disrupted plasminogen/plasmin system inhibiting fibrinolysis (53, 54).

## Therapeutic strategies

**Figure 2:**
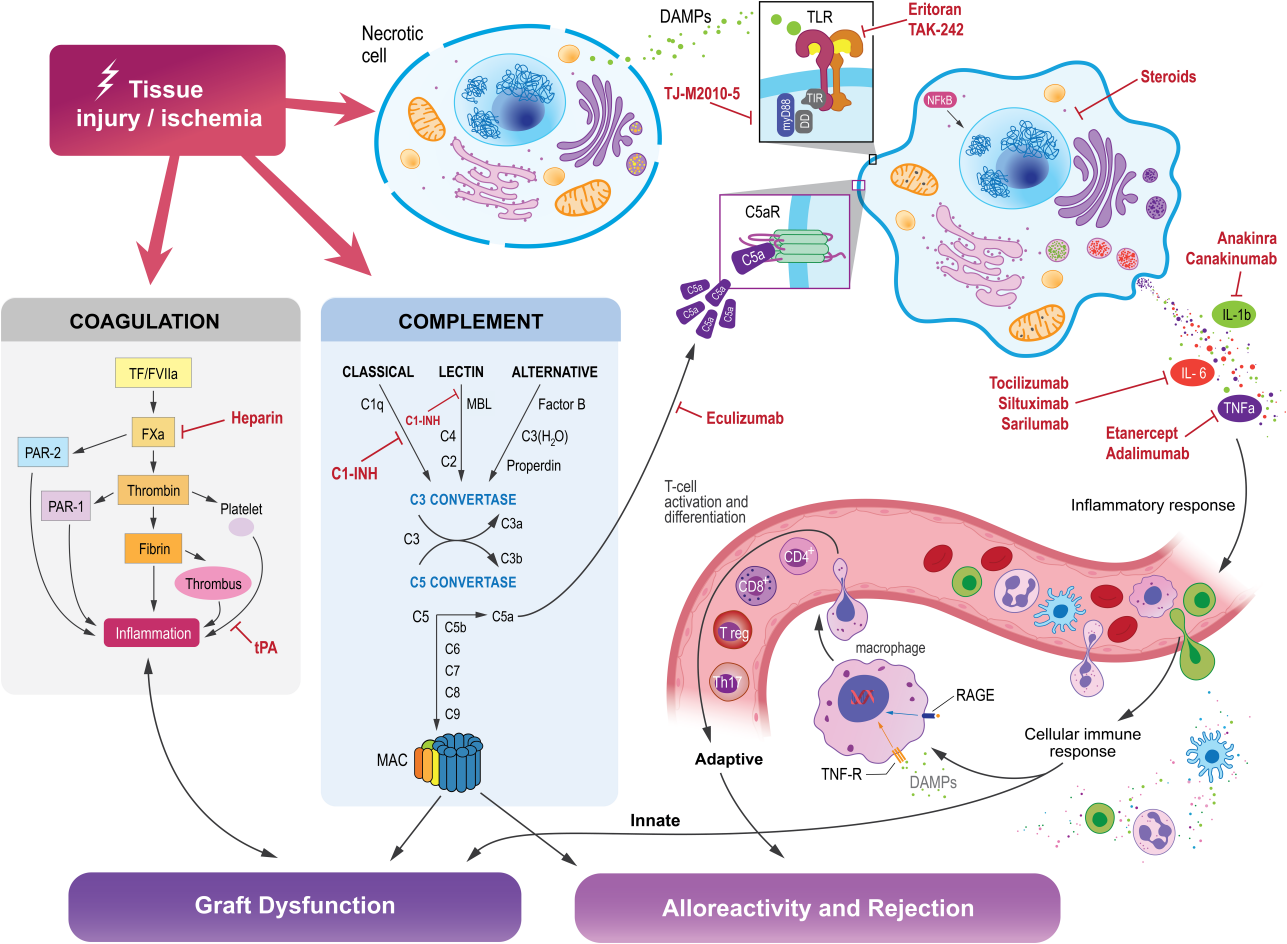
Tissue injury and ischemia activates the innate immune system, which is comprised of intricately interconnected pathways that influence the activation of the adaptive immune system, which can ultimately lead to graft dysfunction, alloreactivity, and rejection in organ transplantation. Molecules within each of these pathways may be targeted to suppress those reactions and promote transplant organ function and acceptance by the host immune system.

Many current therapies in transplantation target the adaptive immune system in order to dampen the immune response to the new organ. The adaptive immune system and innate immune system are intimately linked, and the various components of the innate immune system may serve as novel therapeutic targets (Figure 2).

### Complement inhibition

There is increasing experimental and clinical evidence indicating that complement blockade can be an effective therapeutic target for improving outcomes in organ transplantation (55). A range of therapeutic agents, including monoclonal antibodies, small molecules, and small interfering RNA (siRNA) agents, have been developed to inhibit complement cascade activation. For example, the administration of a soluble complement receptor-1 antagonist (sCR1) can inactivate the C3 and C5 convertases, resulting in reduced neutrophil migration into grafts and reduced post-transplantation reperfusion edema in animal models of lung allotransplantation (56). In clinical trials, sCR1 has been shown to effectively inhibit complement activation, and treating recipient mice with a C5aR antagonist before transplantation has been shown to improve graft survival and reduce monocyte/macrophage infiltration (57, 58).

Eculizumab, a humanized monoclonal antibody that binds to C5 and inhibits its cleavage to C5a, has been approved by the FDA to block complement cascade activation and constitutes another approach to attenuating IRI in organ transplantation (59, 60). Clinical case reports have shown promising results for eculizumab in patients with atypical hemolytic uremic syndrome undergoing kidney transplantation (61). Another potential strategy to inhibit complement cascade activation is to silence C3 using siRNA. In a mouse model of kidney IRI, the systemic administration of C3-specific siRNA reduced renal injury and mouse mortality by diminishing renal C3 synthesis, as C3 is the central component of complement cascade activation where all three pathways converge (62, 63). Silencing the C5a receptor (C5aR) gene using siRNA before the induction of ischemia resulted in reduced expression of the proinflammatory cytokine TNF-α and chemokines MIP-2 and KC, resulting in reductions in neutrophil influx and cell necrosis in the kidneys (64).

In a preclinical context, C1 inhibitors (C1 INH), which are approved for hereditary angioedema treatment, can prevent the activation of both the classical and lectin pathways (65). Research using a brain-dead rat model has shown that C1 INH can mitigate complement activation resulting from brain death (66). Our preliminary studies using a mouse model of renal IRI showed that pre-treating animals with C1 INH led to enhanced renal function, improved survival rates, and decreased C5a release, C3b deposition, as well as neutrophil/macrophage infiltration in the grafts. Furthermore, we observed a significant reduction in tissue fibrosis and TGF-β1 levels 30 and 90 days post-ischemia (67). Utilizing a nonhuman primate kidney transplant model, we demonstrated that managing brain-dead donors with recombinant human C1 INH and heparin substantially diminished complement pathway activation, attenuated systemic inflammatory response, and crucially, averted DGF in all instances compared to controls (68). A subsequent study suggested that C1 INH, when administered systemically to either the brain-dead donor or recipient only, could decrease complement deposition on biopsy and prevent DGF (69). Lastly, in a pig kidney auto-transplant model, C1 INH treatment expedited the recovery of glomerular function and significantly lessened long-term graft fibrosis (70, 71).

Clinical trials pertaining to kidney transplantation have thus far explored the use of C1 and C5 inhibitors as well as a C3 inhibitor. The primary objective of these trials was to avert early graft failure due to DGF, and to prevent both early and late rejection episodes, particularly antibody-mediated rejection (ABMR). Currently, only one phase I/II trial (NCT02134314) comparing the use of the C1-esterase with a placebo has been completed. Although there were no significant differences between groups in the incidence of DGF, the duration of dialysis was notably shortened in the C1-esterase inhibitor group (72). Furthermore, in a follow-up study conducted 3.5 years post-transplantation, a significantly improved estimated glomerular filtration rate (eGFR) was found in the C1-esterase inhibitor group (73).

Recent alternative anti-complement therapies have shown promise by directly administering complement inhibitors to donor kidneys *ex vivo* before transplantation. This method focuses on local kidney complement inhibition, minimizing the impact on the recipient's systemic complement pool. Mirococept, derived from human CR1, was tested in the EMPIRIKAL trial to evaluate its effect on DGF duration. The initial trial demonstrated the feasibility and safety of *ex vivo* Mirococept delivery to kidney allografts and highlighted the need for dose calibration (74). Subsequent pig kidney studies showed strong localization to the treated kidney's tubular epithelium and capillaries, with minimal recipient release. The EMPIRIKAL trial is now revising its human study protocol based on the optimal higher dose achieved in pigs (EMPIRIKAL-2).

### TLR inhibition

Inhibitors and antagonists that target TLR signaling or downstream components have the potential to reduce IRI and improve allograft survival, and several studies on animal models suggest that blocking antibodies against TLR pathway molecules can be an effective therapeutic strategy. For example, the administration of an anti-HMGB1 neutralizing antibody reduced kidney IRI in mice, as shown by lower levels of inflammation and apoptosis in tubular epithelial cells, and reduced infiltration of neutrophils and macrophages (75). In addition, in a model of cardiac transplantation, neutralizing HMGB1 resulted in a reduction of inflammatory CD11b + Ly6C high myeloid cells both at the level of the allograft and the spleen (76).

There have been numerous attempts to block the TLR pathway as a strategy to attenuate ischemia-reperfusion injury (IRI) and enhance allograft survival. One such attempt involves the use of eritoran, a TLR4 antagonist that has been tested in transplantation (77). Eritoran is a synthetic structural analog of the lipid A portion of lipopolysaccharide (LPS) that binds to the MD2-TLR4 receptor and terminates MD2/TLR4-mediated signaling. This action blocks LPS from binding at the cell surface and subsequently inhibits the pro-inflammatory signaling cascade that follows (78). In a rat transplantation model, eritoran treatment induced less monocyte infiltration; lower levels of TNF-α, IL-1β, IL-6, and MCP1; and prolonged survival. There is also evidence to suggest that eritoran may block the HMGB1-TLR4 interaction, which could explain its ability to attenuate IRI. In a mouse model of liver IRI, eritoran was shown to inhibit the TLR4-dependent release of HMGB1, indicating a potential mechanism by which eritoran exerts its protective effects (79).

Another TLR-4 inhibitor, TAK-242, is a small compound that mitigates the release of inflammatory cytokines triggered by pathogens, primarily by impeding TLR-4-mediated signaling pathways. Furthermore, it exhibits suppressive effects on the synthesis of nitric oxide (NO) or tumor necrosis factor (TNF)-alpha, specifically when induced by the TLR4-specific ligand LPS (80). A study investigated the effects of TAK-242 on liver transplant viability in a model of swine Maastricht-category-III cardiac death. TAK-242 was administered prior to induction of cardiac death with mRNA and protein levels of TLR4 signaling pathway evaluated at different time points after induction of cardiac death. The results showed that the mRNA and protein levels of important immune and inflammatory response molecules such as TLR4, NF-ϰB, MCP-1, TNF-α, IL-6, ICAM-1, and MPO, increased significantly after cardiac death. However, infusion of TAK-242 one hour before induction blocked the increase of many of these molecules; though notably, the increase of TLR4 levels was not affected by the TAK-242 infusion (81).

### Other potential targets in the innate immune system

#### IL-6 inhibitors

Tocilizumab, a potent antagonist of the interleukin-6 receptor, has shown remarkable efficacy in reducing HLA antibodies in highly sensitized patients awaiting kidney transplantation and those with antibody-mediated rejection. In 2015, Vo et al. conducted a phase I/II open-label trial (NCT01594424) with ten unresponsive end-stage renal disease patients. The six-month treatment regimen with monthly tocilizumab and IVIg administration resulted in a reduction of DSA levels, enabling five patients to undergo kidney transplantation. Biopsies revealed no evidence of antibody-mediated rejection (AMR) six months post-transplant, but two patients who stopped tocilizumab experienced mild AMR a year later. The study suggested that targeting the IL-6 pathway could offer an alternative for desensitization (82).

A subsequent study by this group evaluated the use of tocilizumab as a rescue therapy for DSA-positive chronic antibody-mediated rejection (cAMR) and transplant glomerulopathy in patients who had previously failed standard treatments. Monthly administration of tocilizumab yielded an 80% graft survival rate at six years, decreased DSA levels, and stabilized renal function without significant adverse events. Four patients did experience graft loss after discontinuing tocilizumab, suggesting rebound IL-6 pathway signaling may contribute to generation of an alloimmune response (83).

Furthermore, tocilizumab has been studied as a first-line therapy for cAMR in kidney transplantation, resulting in GFR stabilization and a significant decrease in DSA with improved pathology observed at six months. IL-6 blockade may also be a potential treatment option for regulating T-cell alloimmune response in kidney transplantation. A recent randomized-controlled trial found that kidney transplant recipients who received tocilizumab had increased T-reg frequency and a blunted T-effector cytokine response (84). A multicenter phase II clinical trial investigating the efficacy of tocilizumab in cardiac transplantation is currently under way to evaluate the impact of tocilizumab on acute and chronic cardiac allograft rejection (NCT03644667).

#### TNF and IL-1 receptor antagonists

The use of anti-inflammatory agents to counteract the harmful effects of proinflammatory cytokines has been extensively investigated, primarily in islet transplantation. Anti-cytokine strategies, such as TNF-alpha inhibition and IL-1 beta antagonism, have demonstrated promising results in improving islet cell function and promoting insulin-independence following transplantation.

TNF-a inhibitors have shown reduced inflammatory markers in animal models and long-lasting efficacy in treating autoimmune disorders (85, 86). The pharmacological characteristics of TNF inhibitors can vary considerably, with Etanercept (ETA) binding and neutralizing lymphotoxin-α3 and lymphotoxin-α2β1, while Infliximab (INF) does not possess these properties (87, 88). A recent qualitative analysis of anti-inflammatory agents in clinical islet transplantation summarized findings from multiple studies evaluating Etanercept monotherapy in allo-islet cell transplantation protocols (89). Although the studies differed in insulin independence (II) definitions and assessment time points, the overall results suggest that Etanercept may contribute to long-term maintenance of II. Despite some inconsistencies in outcomes, an Etanercept-based protocol could be associated with lower islet equivalent (IEQ) requirements (90, 91).

A single prospective, randomized study examined Infliximab as an adjunct to the Edmonton protocol, but no significant clinical benefits or substantial differences were observed in oral glucose tolerance test (OGTT) or intravenous glucose tolerance test (IVGTT) outcomes (92).

IL-1β, a pro-inflammatory cytokine, has been linked to β-cell destruction and diabetes development (93). Interleukin-1 receptor antagonists (IL-1RA) have been demonstrated to effectively counteract the effects of proinflammatory cytokines, including nitric oxide production, necrosis, apoptosis, glucose-stimulated insulin secretion, and mitochondrial dysfunction (94). Several studies have shown that interleukin-1 receptor antagonist (IL-1RA) can improve pancreatic islet transplantation outcomes in mice, primates, and humans (95, 96). In two human trials, anakinra combined with other immunosuppressive agents during the first 1–2 weeks post-transplantation resulted in better engraftment without increased infection risk (97, 98).

Limited data exist on IL-1 beta-directed therapies in transplantation outside the islet field. In renal transplantation, the safety and efficacy of combining IL-1 beta-directed therapy with standard immunosuppressive treatment were evaluated in three patients, with no significant complications except for minor infections (99). Case reports and series indicate that anakinra can be safely used alongside immunosuppressive agents in renal transplant recipients with IL-1 driven diseases, although one patient experienced neutropenia and decreased allograft function, with an unclear link to IL-1 blockade (100).

#### Coagulation cascade inhibition

The coagulation system, which regulates bleeding and clotting, also plays an important role in innate immunity; this, and its interaction with the fibrinolytic system influences IRI. Targeting coagulation factors is complicated by their presence within fibrin clots, often protecting them from inhibitors (45). Therapies such as rapamycin can mitigate fibrinolysis disruption by reducing plasminogen activator inhibitor type 1 (PAI-1) expression, slowing the progression of interstitial fibrosis (101). Furthermore, in porcine models of renal autotransplant, the use of Xa and dual Xa/IIa inhibitors has been shown to be beneficial to graft outcome (102, 103). In addition to inhibiting the complement system, C1 INH has also been shown to inhibit enzymes in the coagulation and fibrinolytic systems, including factor XII and plasmin, respectively (104). As discussed above, in pre-clinical models and ongoing clinical studies, C1 INH has demonstrated a beneficial effect on graft function.

#### Steroids

Glucocorticoids (GCs) play a crucial role in immunosuppression following renal transplantation by mitigating inflammatory responses and leukocyte infiltration. They also facilitate the resolution of inflammation through the suppression of vascular permeability, leukocyte distribution/trafficking, and modulation of cellular death/survival and differentiation processes (105, 106). While GCs were initially believed to exert their anti-inflammatory effects through the inhibition of pro-inflammatory cytokine-encoding gene regulators, recent studies have uncovered alternative mechanisms, including negative interference with inflammatory mediator synthesis, suppression of immune cell activation, and cooperation between the glucocorticoid receptor and transcription factors to induce anti-inflammatory genes (107).

GCs are known to induce neutrophilic leukocytosis by promoting neutrophil maturation and mobilization, an effect that can be blocked by L-selectin adhesion protein inhibition. They also hinder the entire neutrophil activation process, including respiratory burst-related enzyme expression, chemotaxis, phagocytosis, and cytokine secretion. In neutrophils, GCs suppress transcription factors related to pro- and anti-inflammatory genes, leading to upregulated expression of interleukin and pro-inflammatory leukotriene receptors and reduced apoptosis sensitivity, thus extending neutrophil lifespan. Furthermore, endogenous glucocorticoids under stress conditions affect natural killer (NK) cells by reducing their cytolytic activity and inducing pro-inflammatory cytokine synthesis through an epigenetic mechanism, enhancing the expression of IL-6 and INF-γ and promoting histone acetylation in their enhancer regions.

GCs also exhibit direct effects on innate immune cells, as demonstrated by *in vitro* studies on methylprednisolone-treated monocytes, which show increased anti-inflammatory cytokine expression and reduced antigen-presenting capabilities (108, 109). In vivo data from renal transplant recipients treated with methylprednisolone reveal alterations in monocyte populations and TLR4 downregulation on GC-treated monocytes, a critical component in monocyte activation during sepsis and the immune response to transplanted organs (110, 111). Steroids also impact dendritic cell differentiation and maturation, with dexamethasone-exposed monocyte-derived dendritic cells displaying decreased expression of specific markers, impaired antigen-presenting cell function, and reduced cytokine secretion (112).

The anti-inflammatory effect of steroids in transplantation has been widely investigated in the deceased donor setting and initial studies have not consistently show benefits of donor methylprednisolone treatment in transplantation outcome (113). In addition, a large-scale, randomized, double-blind trials in kidney transplantation found that while methylprednisolone pretreatment improved inflammatory and apoptotic gene expression profiles, it did not impact delayed graft function or serum creatinine decline (114, 115). Therefore, high-dose methylprednisolone before organ recovery is not recommended for kidney transplantation.

Although the trial's results were inconclusive, preliminary evidence suggests potential benefits of donor steroid treatment in liver transplantation, prompting continued research efforts into the routine use of steroids in combined hormonal resuscitation protocols (116).

#### Myeloid differentiation factor 88 (MyD88)

Myeloid differentiation factor 88 (MyD88) is a protein that plays a role in signal transduction through TLRs, prompting the production of pro-inflammatory cytokines that promote leukocyte recruitment. Specifically, MyD88 links the IL-1 receptor or other TLR family members to IL-1R-associated kinase (IRAK) kinases that leads to downstream events including NFkB activation and MAP kinase activation (117). Thus, it has a far-reaching effect on the inflammatory response of the immune system.

TJ-M2010-5 is a MyD88 inhibitor that targets and interacts with the MyD88 TIR domain, interfering with dimerization. This molecule has been shown in murine models to play a role in slowing liver fibrosis, and decreasing myocardial ischemic reperfusion injury (118), cerebral reperfusion injury (119), and hepatic reperfusion injury (120). Alleviation of hepatic reperfusion injury with this molecule has been particularly successful when coupled with hepatic hypothermic oxygenated perfusion (121). While these studies have been performed using animal models, they demonstrate some significant promise as a viable therapeutic option for the prevention or reduction of ischemia reperfusion injury in human transplantation.

#### Resveratrol

Resveratrol is a naturally-occurring phenol produced by plants that has been shown to play a role in inflammatory disease prevention and progression. Resveratrol is thought to be produced in response to oxidative stress, and several studies have shown it is able to regulate hepatic metabolism of lipids hence improving metabolic lipid homeostasis and slowing the progression of fatty liver disease (122). Resveratrol also functions as an anti-oxidant, and its main effect is linked to the reduction of reactive nitrogen species, direct elimination of free radicals, improvement in antioxidant enzymatic activity and promotion of synthesis of antioxidant.

Resveratrol interacts with several different immunomodulatory targets including Sirtuins. Sirtuins are NAD+-dependent histone deacetylases, and they are thought play a significant role in immunomodulatory responses and are of significant interest as targets to treat autoimmune disease (123). They are currently studied for their role in promoting organ transplant tolerance, in particular SIRT1 inhibition, which may affect Th17/Treg cell balance (124, 125).

Overall Resveratrol has the potential to play a crucial role in prevention and treatment of liver disease such as anti-oxidant, steatosis and immune-modulator. Additional studies are ongoing with the goal of highlighting efficacy and safety.

#### Vitamin D

Vitamin D plays an important role in the regulation of the immune system, and its deficiency has been shown to play a role in immune dysregulation, particularly in the context of autoimmunity (126). Renal failure can lead to disruption of vitamin D metabolism. In fact, studies have shown that vitamin D deficiency may be a risk factor for rejection or decreased graft function (127–129). As such, studies have demonstrated that vitamin D supplementation may serve as a means to promote immunity and maintain graft function (130).

## Conclusion

In order for the immune system to function appropriately, there must be a complex synergy between the innate and adaptive arms. The innate immune system is essential for the highly specialized adaptive immune system to function. Furthermore, modulation of this complex system is essential for successful organ transplantation, particularly to prevent ischemia reperfusion injury and rejection, and to promote graft function. There is an abundance of research that highlights components of the innate immune system, their role in transplantation, and how those components may be harnessed for therapeutic benefit. Ongoing studies are identifying novel therapeutic targets within the innate immune system that have the potential to greatly impact management of transplant patients.

## References

[1] Delves PJ Roitt IM. The immune system. First of two parts. N Engl J Med. (2000) 343(1):37–49. 10.1056/NEJM200007063430107 10882768

[2] Medzhitov R. Origin and physiological roles of inflammation. Nature. (2008) 454(7203):428–35. 10.1038/nature07201 18650913

[3] Wynn TA Ramalingam TR. Mechanisms of fibrosis: therapeutic translation for fibrotic disease. Nat Med. (2012) 18(7):1028–40. 10.1038/nm.2807 22772564 PMC3405917

[4] Medzhitov R. Toll-like receptors and innate immunity. Nat Rev Immunol. (2001) 1(2):135–45. 10.1038/35100529 11905821

[5] Takeda K Akira S. Toll-like receptors in innate immunity. Int Immunol. (2005) 17(1):1–14. 10.1093/intimm/dxh186 15585605

[6] Bonilla FA Oettgen HC. Adaptive immunity. J Allergy Clin Immunol. (2010) 125(2 Suppl 2):S33–40. 10.1016/j.jaci.2009.09.017 20061006

[7] Jain A Pasare C. Innate control of adaptive immunity: beyond the three-signal paradigm. J Immunol. (2017) 198(10):3791–800. 10.4049/jimmunol.1602000 28483987 PMC5442885

[8] Iwasaki A Medzhitov R. Control of adaptive immunity by the innate immune system. Nat Immunol. (2015) 16(4):343–53. 10.1038/ni.3123 25789684 PMC4507498

[9] Ricklin D Hajishengallis G Yang K Lambris JD. Complement: a key system for immune surveillance and homeostasis. Nat Immunol. (2010) 11(9):785–97. 10.1038/ni.1923 20720586 PMC2924908

[10] Asgari E Zhou W Sacks S. Complement in organ transplantation. Curr Opin Organ Transplant. (2010) 15(4):486–91. 10.1097/MOT.0b013e32833b9cb7 20631616 PMC3100572

[11] Sacks S Lee Q Wong W Zhou W. The role of complement in regulating the alloresponse. Curr Opin Organ Transplant. (2009) 14(1):10–5. 10.1097/MOT.0b013e32831ec551 19337140

[12] Sacks SH Zhou W. The role of complement in the early immune response to transplantation. Nat Rev Immunol. (2012) 12(6):431–42. 10.1038/nri3225 22627861

[13] de Groot H Rauen U. Ischemia-reperfusion injury: processes in pathogenetic networks: a review. Transplant Proc. (2007) 39(2):481–4. 10.1016/j.transproceed.2006.12.012 17362763

[14] Hepburn NJ Ruseva MM Harris CL Morgan BP. Complement, roles in renal disease and modulation for therapy. Clin Nephrol. (2008) 70(5):357–76. 10.5414/CNP70357 19000536

[15] McCullough JW Renner B Thurman JM. The role of the complement system in acute kidney injury. Semin Nephrol. (2013) 33(6):543–56. 10.1016/j.semnephrol.2013.08.005 24161039 PMC3816009

[16] Ricklin D Lambris JD. Complement in immune and inflammatory disorders: pathophysiological mechanisms. J Immunol. (2013) 190(8):3831–8. 10.4049/jimmunol.1203487 23564577 PMC3623009

[17] Peng Q Li K Smyth LA Xing G Wang N Meader L C3a and C5a promote renal ischemia-reperfusion injury. J Am Soc Nephrol. (2012) 23(9):1474–85. 10.1681/ASN.2011111072 22797180 PMC3431410

[18] Arumugam T Okun E Tang S Thundyil J Taylor S Woodruff T. Toll-like receptors in ischemia-reperfusion injury. Shock. (2009) 32(1):4–16. 10.1097/SHK.0b013e318193e333 19008778

[19] Zhang Q Raoof M Chen Y Sumi Y Sursal T Junger W Circulating mitochondrial DAMPs cause inflammatory responses to injury. Nature. (2010) 464(7285):104–7. 10.1038/nature08780 20203610 PMC2843437

[20] Sims GP Rowe DC Rietdijk ST Herbst R Coyle AJ. HMGB1 and RAGE in inflammation and cancer. Annu Rev Immunol. (2010) 28:367–88. 10.1146/annurev.immunol.021908.132603 20192808

[21] Anders HJ Schaefer L. Beyond tissue injury-damage-associated molecular patterns, toll-like receptors, and inflammasomes also drive regeneration and fibrosis. J Am Soc Nephrol. (2014) 25(7):1387–400. 10.1681/ASN.2014010117 24762401 PMC4073442

[22] Bonventre JV Zuk A. Ischemic acute renal failure: an inflammatory disease? Kidney Int. (2004) 66(2):480–5. 10.1111/j.1523-1755.2004.761_2.x 15253693

[23] Patel NS Chatterjee PK Di Paola R Mazzon E Britti D De Sarro A Endogenous interleukin-6 enhances the renal injury, dysfunction, and inflammation caused by ischemia/reperfusion. J Pharmacol Exp Ther. (2005) 312(3):1170–8. 10.1124/jpet.104.078659 15572648

[24] Zhang J Li Q Zou YR Wu SK Lu XH Li GS HMGB1-TLR4-IL-23-IL-17A axis accelerates renal ischemia-reperfusion injury via the recruitment and migration of neutrophils. Int Immunopharmacol. (2021) 94:107433. 10.1016/j.intimp.2021.107433 33592404

[25] Kurihara C Lecuona E Wu Q Yang W Núñez-Santana FL Akbarpour M Crosstalk between nonclassical monocytes and alveolar macrophages mediates transplant ischemia-reperfusion injury through classical monocyte recruitment. JCI Insight. (2021) 6(6). 10.1172/jci.insight.147282 33621212 PMC8026186

[26] Yin E Fukuhara T Takeda K Kojima Y Fukuhara K Ikejima K Anti-CD321 antibody immunotherapy protects liver against ischemia and reperfusion-induced injury. Sci Rep. (2021) 11(1):6312. 10.1038/s41598-021-85001-2 33737554 PMC7973783

[27] Miller CL Madsen JC. IL-6 directed therapy in transplantation. Curr Transplant Rep. (2021) 8(3):191–204. 10.1007/s40472-021-00331-4 34099967 PMC8173333

[28] Watts RP Thom O Fraser JF. Inflammatory signalling associated with brain dead organ donation: from brain injury to brain stem death and posttransplant ischaemia reperfusion injury. J Transplant. (2013) 2013:521369. 10.1155/2013/521369 23691272 PMC3649190

[29] Zitur LJ Chlebeck PJ Odorico SK Danobeitia JS Zens TJ Van Kooten C Brain death enhances activation of the innate immune system and leads to reduced renal metabolic gene expression. Transplantation. (2019) 103(9):1821–33. 10.1097/TP.0000000000002744 30964836 PMC6713605

[30] Jurewicz M Takakura A Augello A Movahedi Naini S Ichimura T Zandi-Nejad K Ischemic injury enhances dendritic cell immunogenicity via TLR4 and NF-kappa B activation. J Immunol. (2010) 184(6):2939–48. 10.4049/jimmunol.0901889 20164431

[31] Romano M Sironi M Toniatti C Polentarutti N Fruscella P Ghezzi P Role of IL-6 and its soluble receptor in induction of chemokines and leukocyte recruitment. Immunity. (1997) 6(3):315–25. 10.1016/S1074-7613(00)80334-9 9075932

[32] Uehara M Solhjou Z Banouni N Kasinath V Xiaqun Y Dai L Ischemia augments alloimmune injury through IL-6-driven CD4. Sci Rep. (2018) 8(1):2461. 10.1038/s41598-018-20858-4 29410442 PMC5802749

[33] Raasveld MH Weening JJ Kerst JM Surachno S ten Berge RJ. Local production of interleukin-6 during acute rejection in human renal allografts. Nephrol Dial Transplant. (1993) 8(1):75–8. 10.1093/oxfordjournals.ndt.a092278 8381942

[34] Waiser J Budde K Katalinic A Kuerzdörfer M Riess R Neumayer HH. Interleukin-6 expression after renal transplantation. Nephrol Dial Transplant. (1997) 12(4):753–9. 10.1093/ndt/12.4.753 9141007

[35] Shen H Goldstein DR. IL-6 and TNF-alpha synergistically inhibit allograft acceptance. J Am Soc Nephrol. (2009) 20(5):1032–40. 10.1681/ASN.2008070778 19357252 PMC2678042

[36] Kim I Wu G Chai NN Klein AS Jordan S. Anti-interleukin 6 receptor antibodies attenuate antibody recall responses in a mouse model of allosensitization. Transplantation. (2014) 98(12):1262–70. 10.1097/TP.0000000000000437 25286051

[37] Wu G Chai N Kim I Klein AS Jordan SC. Monoclonal anti-interleukin-6 receptor antibody attenuates donor-specific antibody responses in a mouse model of allosensitization. Transpl Immunol. (2013) 28(2-3):138–43. 10.1016/j.trim.2013.03.003 23562586

[38] Fogal B Yi T Wang C Rao DA Lebastchi A Kulkarni S Neutralizing IL-6 reduces human arterial allograft rejection by allowing emergence of CD161+ CD4+ regulatory T cells. J Immunol. (2011) 187(12):6268–80. 10.4049/jimmunol.1003774 22084439 PMC3237826

[39] Liang Y Christopher K Finn PW Colson YL Perkins DL. Graft produced interleukin-6 functions as a danger signal and promotes rejection after transplantation. Transplantation. (2007) 84(6):771–7. 10.1097/01.tp.0000281384.24333.0b 17893611

[40] Booth AJ Grabauskiene S Wood SC Lu G Burrell BE Bishop DK. IL-6 promotes cardiac graft rejection mediated by CD4+ cells. J Immunol. (2011) 187(11):5764–71. 10.4049/jimmunol.1100766 22025555 PMC3221839

[41] Zhao X Boenisch O Yeung M Mfarrej B Yang S Turka LA Critical role of proinflammatory cytokine IL-6 in allograft rejection and tolerance. Am J Transplant. (2012) 12(1):90–101. 10.1111/j.1600-6143.2011.03770.x 21992708

[42] Krams SM Falco DA Villanueva JC Rabkin J Tomlanovich SJ Vincenti F Cytokine and T cell receptor gene expression at the site of allograft rejection. Transplantation. (1992) 53(1):151–6. 10.1097/00007890-199201000-00031 1733064

[43] Gorczynski RM Adams RB Levy GA Chung SW. Correlation of peripheral blood lymphocyte and intragraft cytokine mRNA expression with rejection in orthotopic liver transplantation. Surgery. (1996) 120(3):496–502. 10.1016/S0039-6060(96)80069-9 8784403

[44] Kita Y Iwaki Y Demetris AJ Starzl TE. Evaluation of sequential serum interleukin-6 levels in liver allograft recipients. Transplantation. (1994) 57(7):1037–41. 10.1097/00007890-199404150-00009 8165699 PMC3022505

[45] Stallone G Pontrelli P Rascio F Castellano G Gesualdo L Grandaliano G. Coagulation and fibrinolysis in kidney graft rejection. Front Immunol. (2020) 11:1807. 10.3389/fimmu.2020.01807 32983089 PMC7477357

[46] Pawlicki J Cierpka L Król R Ziaja J. Analysis of coagulation parameters in the early period after kidney transplantation. Transplant Proc. (2007) 39(9):2754–5. 10.1016/j.transproceed.2007.08.050 18021978

[47] Thiagarajan RR Winn RK Harlan JM. The role of leukocyte and endothelial adhesion molecules in ischemia-reperfusion injury. Thromb Haemost. (1997) 78(1):310–4. 10.1055/s-0038-1657545 9198172

[48] Pontrelli P Cariello M Rascio F Gigante M Verrienti R Tataranni T Thrombin may modulate dendritic cell activation in kidney transplant recipients with delayed graft function. Nephrol Dial Transplant. (2015) 30(9):1480–7. 10.1093/ndt/gfv129 26056176

[49] Grandaliano G Di Paolo S Monno R Stallone G Ranieri E Pontrelli P Protease-activated receptor 1 and plasminogen activator inhibitor 1 expression in chronic allograft nephropathy: the role of coagulation and fibrinolysis in renal graft fibrosis. Transplantation. (2001) 72(8):1437–43. 10.1097/00007890-200110270-00018 11685117

[50] Manook M Kwun J Sacks S Dorling A Mamode N Knechtle S. Innate networking: thrombotic microangiopathy, the activation of coagulation and complement in the sensitized kidney transplant recipient. Transplant Rev (Orlando). (2018) 32(3):119–26. 10.1016/j.trre.2018.01.001 29935708 PMC6497150

[51] Bronisz M Rość D Bronisz A Manitius J Nartowicz E. The role of intrinsic fibrinolytic system activation in pathogenesis of hemostasis disturbances in hemodialyzed patients with chronic renal failure. Ren Fail. (2004) 26(3):223–9. 10.1081/JDI-120039519 15354969

[52] Ziętek Z Iwan-Ziętek I Sulikowski T Sieńko J Zukowski M Kaczmarczyk M The effect of cause of cadaveric kidney donors death on fibrinolysis and blood coagulation processes. Transplant Proc. (2011) 43(8):2866–70. 10.1016/j.transproceed.2011.08.011 21996175

[53] Roelofs JJ Rowshani AT van den Berg JG Claessen N Aten J ten Berge IJ Expression of urokinase plasminogen activator and its receptor during acute renal allograft rejection. Kidney Int. (2003) 64(5):1845–53. 10.1046/j.1523-1755.2003.00261.x 14531820

[54] Staniszewska M Dziedziejko V Kwiatkowska E Tkacz M Puchałowicz K Safranow K Plasma concentration of urokinase plasminogen activator receptor is a marker of kidney allograft function. Ir J Med Sci. (2018) 187(4):1083–7. 10.1007/s11845-018-1767-4 29497975

[55] Grafals M Thurman JM. The role of complement in organ transplantation. Front Immunol. (2019) 10:2380. 10.3389/fimmu.2019.02380 31636644 PMC6788431

[56] Schmid RA Zollinger A Singer T Hillinger S Leon-Wyss JR Schöb OM Effect of soluble complement receptor type 1 on reperfusion edema and neutrophil migration after lung allotransplantation in swine. J Thorac Cardiovasc Surg. (1998) 116(1):90–7. 10.1016/S0022-5223(98)70246-6 9671902

[57] Keshavjee S Davis RD Zamora MR de Perrot M Patterson GA. A randomized, placebo-controlled trial of complement inhibition in ischemia-reperfusion injury after lung transplantation in human beings. J Thorac Cardiovasc Surg. (2005) 129(2):423–8. 10.1016/j.jtcvs.2004.06.048 15678055

[58] Gueler F Rong S Gwinner W Mengel M Bröcker V Schön S Complement 5a receptor inhibition improves renal allograft survival. J Am Soc Nephrol. (2008) 19(12):2302–12. 10.1681/ASN.2007111267 18753257 PMC2588101

[59] Kaabak M Babenko N Shapiro R Zokoyev A Dymova O Kim E. A prospective randomized, controlled trial of eculizumab to prevent ischemia-reperfusion injury in pediatric kidney transplantation. Pediatr Transplant. (2018) 22(2). 10.1111/petr.13129 29377474

[60] Grenda R Durlik M. Eculizumab in renal transplantation: a 2017 update. Ann Transplant. (2017) 22:550–4. 10.12659/AOT.905917 28894081 PMC12574081

[61] Favi E Molinari P Alfieri C Castellano G Ferraresso M Cresseri D. Case report: eculizumab plus obinutuzumab induction in a deceased donor kidney transplant recipient with DEAP-HUS. Front Immunol. (2022) 13:1073808. 10.3389/fimmu.2022.1073808 36591301 PMC9795842

[62] Zheng X Feng B Chen G Zhang X Li M Sun H Preventing renal ischemia-reperfusion injury using small interfering RNA by targeting complement 3 gene. Am J Transplant. (2006) 6(9):2099–108. 10.1111/j.1600-6143.2006.01427.x 16796725

[63] Zheng X Zhang X Sun H Feng B Li M Chen G Protection of renal ischemia injury using combination gene silencing of complement 3 and caspase 3 genes. Transplantation. (2006) 82(12):1781–6. 10.1097/01.tp.0000250769.86623.a3 17198276

[64] Zheng X Zhang X Feng B Sun H Suzuki M Ichim T Gene silencing of complement C5a receptor using siRNA for preventing ischemia/reperfusion injury. Am J Pathol. (2008) 173(4):973–80. 10.2353/ajpath.2008.080103 18772341 PMC2543066

[65] Davis AE. Biological effects of C1 inhibitor. Drug News Perspect. (2004) 17(7):439–46. 10.1358/dnp.2004.17.7.863703 15514703

[66] Poppelaars F Jager NM Kotimaa J Leuvenink HGD Daha MR van Kooten C C1-Inhibitor treatment decreases renal injury in an established brain-dead rat model. Transplantation. (2018) 102(1):79–87. 10.1097/TP.0000000000001895 28731906

[67] Danobeitia JS Ziemelis M Ma X Zitur LJ Zens T Chlebeck PJ Complement inhibition attenuates acute kidney injury after ischemia-reperfusion and limits progression to renal fibrosis in mice. PLoS One. (2017) 12(8):e0183701. 10.1371/journal.pone.0183701 28832655 PMC5568291

[68] Danobeitia JS Zens TJ Chlebeck PJ Zitur LJ Reyes JA Eerhart MJ Targeted donor complement blockade after brain death prevents delayed graft function in a nonhuman primate model of kidney transplantation. Am J Transplant. (2020) 20(6):1513–26. 10.1111/ajt.15777 31922336 PMC7261643

[69] Eerhart MJ Reyes JA Blanton CL Danobeitia JS Chlebeck PJ Zitur LJ Complement blockade in recipients prevents delayed graft function and delays antibody-mediated rejection in a nonhuman primate model of kidney transplantation. Transplantation. (2022) 106(1):60–71. 10.1097/TP.0000000000003754 34905763 PMC8674492

[70] Castellano G Melchiorre R Loverre A Ditonno P Montinaro V Rossini M Therapeutic targeting of classical and lectin pathways of complement protects from ischemia-reperfusion-induced renal damage. Am J Pathol. (2010) 176(4):1648–59. 10.2353/ajpath.2010.090276 20150432 PMC2843457

[71] Castellano G Intini A Stasi A Divella C Gigante M Pontrelli P Complement modulation of anti-aging factor klotho in ischemia/reperfusion injury and delayed graft function. Am J Transplant. (2016) 16(1):325–33. 10.1111/ajt.13415 26280899

[72] Jordan SC Choi J Aubert O Haas M Loupy A Huang E A phase I/II, double-blind, placebo-controlled study assessing safety and efficacy of C1 esterase inhibitor for prevention of delayed graft function in deceased donor kidney transplant recipients. Am J Transplant. (2018) 18(12):2955–64. 10.1111/ajt.14767 29637714

[73] Huang E Vo A Choi J Ammerman N Lim K Sethi S Three-year outcomes of a randomized, double-blind, placebo-controlled study assessing safety and efficacy of C1 esterase inhibitor for prevention of delayed graft function in deceased donor kidney transplant recipients. Clin J Am Soc Nephrol. (2020) 15(1):109–16. 10.2215/CJN.04840419 31843975 PMC6946080

[74] Kassimatis T Qasem A Douiri A Ryan EG Rebollo-Mesa I Nichols LL A double-blind randomised controlled investigation into the efficacy of mirococept (APT070) for preventing ischaemia reperfusion injury in the kidney allograft (EMPIRIKAL): study protocol for a randomised controlled trial. Trials. (2017) 18(1):255. 10.1186/s13063-017-1972-x 28587616 PMC5461672

[75] Wu H Ma J Wang P Corpuz TM Panchapakesan U Wyburn KR HMGB1 contributes to kidney ischemia reperfusion injury. J Am Soc Nephrol. (2010) 21(11):1878–90. 10.1681/ASN.2009101048 20847143 PMC3014003

[76] Zou H Yang Y Gao M Zhang B Ming B Sun Y HMGB1 Is involved in chronic rejection of cardiac allograft via promoting inflammatory-like mDCs. Am J Transplant. (2014) 14(8):1765–77. 10.1111/ajt.12781 24984831

[77] Liu M Gu M Xu D Lv Q Zhang W Wu Y. Protective effects of toll-like receptor 4 inhibitor eritoran on renal ischemia-reperfusion injury. Transplant Proc. (2010) 42(5):1539–44. 10.1016/j.transproceed.2010.03.133 20620471

[78] Chen F Zou L Williams B Chao W. Targeting toll-like receptors in sepsis: from bench to clinical trials. Antioxid Redox Signal. (2021) 35(15):1324–39. 10.1089/ars.2021.0005 33588628 PMC8817700

[79] Mcdonald KA Huang H Tohme S Loughran P Ferrero K Billiar T Toll-like receptor 4 (TLR4) antagonist eritoran tetrasodium attenuates liver ischemia and reperfusion injury through inhibition of high-mobility group box protein B1 (HMGB1) signaling. Mol Med. (2015) 20(1):639–48. 10.2119/molmed.2014.00076 25375408 PMC4365061

[80] Samarpita S Kim JY Rasool MK Kim KS. Investigation of toll-like receptor (TLR) 4 inhibitor TAK-242 as a new potential anti-rheumatoid arthritis drug. Arthritis Res Ther. (2020) 22(1):16. 10.1186/s13075-020-2097-2 31973752 PMC6979396

[81] Shao Z Jiao B Liu T Cheng Y Liu H Liu Y. TAK-242 treatment ameliorates liver ischemia/reperfusion injury by inhibiting TLR4 signaling pathway in a swine model of Maastricht-category-III cardiac death. Biomed Pharmacother. (2016) 84:495–501. 10.1016/j.biopha.2016.09.036 27685793

[82] Vo AA Choi J Kim I Louie S Cisneros K Kahwaji J A phase I/II trial of the interleukin-6 receptor-specific humanized monoclonal (tocilizumab)+intravenous immunoglobulin in difficult to desensitize patients. Transplantation. (2015) 99(11):2356–63. 10.1097/TP.0000000000000741 26018350

[83] Choi J Aubert O Vo A Loupy A Haas M Puliyanda D Assessment of tocilizumab (anti-interleukin-6 receptor monoclonal) as a potential treatment for chronic antibody-mediated rejection and transplant glomerulopathy in HLA-sensitized renal allograft recipients. Am J Transplant. (2017) 17(9):2381–9. 10.1111/ajt.14228 28199785

[84] Chandran S Leung J Hu C Laszik ZG Tang Q Vincenti FG. Interleukin-6 blockade with tocilizumab increases tregs and reduces T effector cytokines in renal graft inflammation: a randomized controlled trial. Am J Transplant. (2021) 21(7):2543–54. 10.1111/ajt.16459 33331082

[85] Westwell-Roper C Dai DL Soukhatcheva G Potter KJ van Rooijen N Ehses JA IL-1 blockade attenuates islet amyloid polypeptide-induced proinflammatory cytokine release and pancreatic islet graft dysfunction. J Immunol. (2011) 187(5):2755–65. 10.4049/jimmunol.1002854 21813778

[86] Rabinovitch A Sumoski W Rajotte RV Warnock GL. Cytotoxic effects of cytokines on human pancreatic islet cells in monolayer culture. J Clin Endocrinol Metab. (1990) 71(1):152–6. 10.1210/jcem-71-1-152 2115042

[87] Tracey D Klareskog L Sasso EH Salfeld JG Tak PP. Tumor necrosis factor antagonist mechanisms of action: a comprehensive review. Pharmacol Ther. (2008) 117(2):244–79. 10.1016/j.pharmthera.2007.10.001 18155297

[88] Kaymakcalan Z Sakorafas P Bose S Scesney S Xiong L Hanzatian DK Comparisons of affinities, avidities, and complement activation of adalimumab, infliximab, and etanercept in binding to soluble and membrane tumor necrosis factor. Clin Immunol. (2009) 131(2):308–16. 10.1016/j.clim.2009.01.002 19188093

[89] Szempruch KR Banerjee O McCall RC Desai CS. Use of anti-inflammatory agents in clinical islet cell transplants: a qualitative systematic analysis. Islets. (2019) 11(3):65–75. 10.1080/19382014.2019.1601543 31149871 PMC6548473

[90] Faradji RN Tharavanij T Messinger S Froud T Pileggi A Monroy K Long-term insulin independence and improvement in insulin secretion after supplemental islet infusion under exenatide and etanercept. Transplantation. (2008) 86(12):1658–65. 10.1097/TP.0b013e31818fe448 19104401 PMC2759384

[91] Gangemi A Salehi P Hatipoglu B Martellotto J Barbaro B Kuechle JB Islet transplantation for brittle type 1 diabetes: the UIC protocol. Am J Transplant. (2008) 8(6):1250–61. 10.1111/j.1600-6143.2008.02234.x 18444920

[92] Froud T Ricordi C Baidal DA Hafiz MM Ponte G Cure P Islet transplantation in type 1 diabetes mellitus using cultured islets and steroid-free immunosuppression: Miami experience. Am J Transplant. (2005) 5(8):2037–46. 10.1111/j.1600-6143.2005.00957.x 15996257

[93] Yan LL Ye LP Chen YH He SQ Zhang CY Mao XL The influence of microenvironment on survival of intraportal transplanted islets. Front Immunol. (2022) 13:849580. 10.3389/fimmu.2022.849580 35418988 PMC8995531

[94] Schwarznau A Hanson MS Sperger JM Schram BR Danobeitia JS Greenwood KK IL-1beta receptor blockade protects islets against pro-inflammatory cytokine induced necrosis and apoptosis. J Cell Physiol. (2009) 220(2):341–7. 10.1002/jcp.21770 19334038 PMC2890273

[95] Sahraoui A Kloster-Jensen K Ueland T Korsgren O Foss A Scholz H. Anakinra and tocilizumab enhance survival and function of human islets during culture: implications for clinical islet transplantation. Cell Transplant. (2014) 23(10):1199–211. 10.3727/096368913X667529 23635711

[96] Danobeitia JS Hanson MS Chlebeck P Park E Sperger JM Schwarznau A Donor pretreatment with IL-1 receptor antagonist attenuates inflammation and improves functional potency in islets from brain-dead nonhuman primates. Cell Transplant. (2015) 24(9):1863–77. 10.3727/096368914X681045 24759633 PMC5701288

[97] Maffi P Berney T Nano R Niclauss N Bosco D Melzi R Calcineurin inhibitor-free immunosuppressive regimen in type 1 diabetes patients receiving islet transplantation: single-group phase 1/2 trial. Transplantation. (2014) 98(12):1301–9. 10.1097/TP.0000000000000396 25286053

[98] Takita M Matsumoto S Shimoda M Chujo D Itoh T Sorelle JA Safety and tolerability of the T-cell depletion protocol coupled with anakinra and etanercept for clinical islet cell transplantation. Clin Transplant. (2012) 26(5):E471–84. 10.1111/ctr.12011 23061757 PMC4082563

[99] Mulders-Manders CM Baas MC Molenaar FM Simon A. Peri- and postoperative treatment with the interleukin-1 receptor antagonist anakinra is safe in patients undergoing renal transplantation: case series and review of the literature. Front Pharmacol. (2017) 8:342. 10.3389/fphar.2017.00342 28620307 PMC5449651

[100] Direz G Noël N Guyot C Toupance O Salmon JH Eschard JP. Efficacy but side effects of anakinra therapy for chronic refractory gout in a renal transplant recipient with preterminal chronic renal failure. Joint Bone Spine. (2012) 79(6):631. 10.1016/j.jbspin.2012.04.009 22841588

[101] Pontrelli P Rossini M Infante B Stallone G Schena A Loverre A Rapamycin inhibits PAI-1 expression and reduces interstitial fibrosis and glomerulosclerosis in chronic allograft nephropathy. Transplantation. (2008) 85(1):125–34. 10.1097/01.tp.0000296831.91303.9a 18192922

[102] Tillet S Giraud S Delpech PO Thuillier R Ameteau V Goujon JM Kidney graft outcome using an anti-Xa therapeutic strategy in an experimental model of severe ischaemia-reperfusion injury. Br J Surg. (2015) 102(1):132–42; discussion 42. 10.1002/bjs.9662 25402331

[103] Tillet S Giraud S Kerforne T Saint-Yves T Joffrion S Goujon JM Inhibition of coagulation proteases Xa and IIa decreases ischemia-reperfusion injuries in a preclinical renal transplantation model. Transl Res. (2016) 178:95–106.e1. 10.1016/j.trsl.2016.07.014 27513209

[104] Berger M Baldwin WM Jordan SC. Potential roles for C1 inhibitor in transplantation. Transplantation. (2016) 100(7):1415–24. 10.1097/TP.0000000000000995 26599489 PMC7264819

[105] Steiner RW Awdishu L. Steroids in kidney transplant patients. Semin Immunopathol. (2011) 33(2):157–67. 10.1007/s00281-011-0259-7 21331501 PMC3082701

[106] Coutinho AE Chapman KE. The anti-inflammatory and immunosuppressive effects of glucocorticoids, recent developments and mechanistic insights. Mol Cell Endocrinol. (2011) 335(1):2–13. 10.1016/j.mce.2010.04.005 20398732 PMC3047790

[107] Xavier AM Anunciato AK Rosenstock TR Glezer I. Gene expression control by glucocorticoid receptors during innate immune responses. Front Endocrinol (Lausanne). (2016) 7:31. 10.3389/fendo.2016.00031 27148162 PMC4835445

[108] Lee SW Tsou AP Chan H Thomas J Petrie K Eugui EM Glucocorticoids selectively inhibit the transcription of the interleukin 1 beta gene and decrease the stability of interleukin 1 beta mRNA. Proc Natl Acad Sci U S A. (1988) 85(4):1204–8. 10.1073/pnas.85.4.1204 3257575 PMC279735

[109] Debets JM Ruers TJ van der Linden MP van der Linden CJ Buurman WA. Inhibitory effect of corticosteroids on the secretion of tumour necrosis factor (TNF) by monocytes is dependent on the stimulus inducing TNF synthesis. Clin Exp Immunol. (1989) 78(2):224–9. 12412753 PMC1534652

[110] Rogacev KS Zawada AM Hundsdorfer J Achenbach M Held G Fliser D Immunosuppression and monocyte subsets. Nephrol Dial Transplant. (2015) 30(1):143–53. 10.1093/ndt/gfu315 25313167

[111] Kaczorowski DJ Nakao A Mollen KP Vallabhaneni R Sugimoto R Kohmoto J Toll-like receptor 4 mediates the early inflammatory response after cold ischemia/reperfusion. Transplantation. (2007) 84(10):1279–87. 10.1097/01.tp.0000287597.87571.17 18049113

[112] Rozkova D Horvath R Bartunkova J Spisek R. Glucocorticoids severely impair differentiation and antigen presenting function of dendritic cells despite upregulation of toll-like receptors. Clin Immunol. (2006) 120(3):260–71. 10.1016/j.clim.2006.04.567 16765091

[113] D'Aragon F Belley-Cote E Agarwal A Frenette AJ Lamontagne F Guyatt G Effect of corticosteroid administration on neurologically deceased organ donors and transplant recipients: a systematic review and meta-analysis. BMJ Open. (2017) 7(6):e014436. 10.1136/bmjopen-2016-014436 PMC5734295 28667204

[114] Kainz A Wilflingseder J Mitterbauer C Haller M Burghuber C Perco P Steroid pretreatment of organ donors to prevent postischemic renal allograft failure: a randomized, controlled trial. Ann Intern Med. (2010) 153(4):222–30. 10.7326/0003-4819-153-4-201008170-00003 20713790

[115] Reindl-Schwaighofer R Kainz A Jelencsics K Heinzel A Berlakovich G Remport Á Steroid pretreatment of organ donors does not impact on early rejection and long-term kidney allograft survival: results from a multicenter randomized, controlled trial. Am J Transplant. (2019) 19(6):1770–6. 10.1111/ajt.15252 30614649 PMC6563104

[116] Kotsch K Ulrich F Reutzel-Selke A Pascher A Faber W Warnick P Methylprednisolone therapy in deceased donors reduces inflammation in the donor liver and improves outcome after liver transplantation: a prospective randomized controlled trial. Ann Surg. (2008) 248(6):1042–50. 10.1097/SLA.0b013e318190e70c 19092349

[117] Deguine J Barton GM. Myd88: a central player in innate immune signaling. F1000Prime Rep. (2014) 6:97. 10.12703/P6-97 25580251 PMC4229726

[118] Miao Y Ding Z Zou Z Yang Y Yang M Zhang X Inhibition of MyD88 by a novel inhibitor reverses two-thirds of the infarct area in myocardial ischemia and reperfusion injury. Am J Transl Res. (2020) 12(9):5151–69. 33042411 PMC7540094

[119] Li Z Zhao M Zhang X Lu Y Yang Y Xie Y TJ-M2010-5, a novel CNS drug candidate, attenuates acute cerebral ischemia-reperfusion injury through the MyD88/NF-κB and ERK pathway. Front Pharmacol. (2022) 13:1080438. 10.3389/fphar.2022.1080438 36588708 PMC9797592

[120] Xie Y Du D Zhang L Yang Y Zou Z Li Z TJ-M2010-5, a self-developed MyD88 inhibitor, attenuates liver fibrosis by inhibiting the NF-κB pathway. Chem Biol Interact. (2022) 354:109839. 10.1016/j.cbi.2022.109839 35101388

[121] Zhou W Peng S Du P Zhou P Xue C Ye Q. Hypothermic oxygenated perfusion combined with TJ-M2010-5 alleviates hepatic ischemia-reperfusion injury in donation after circulatory death. Int Immunopharmacol. (2022) 105:108541. 10.1016/j.intimp.2022.108541 35063749

[122] Izzo C Annunziata M Melara G Sciorio R Dallio M Masarone M The role of resveratrol in liver disease: a comprehensive review from in vitro to clinical trials. Nutrients. (2021) 13(3). 10.3390/nu13030933 PMC7999728 33805795

[123] Hamaidi I Kim S. Sirtuins are crucial regulators of T cell metabolism and functions. Exp Mol Med. (2022) 54(3):207–15. 10.1038/s12276-022-00739-7 35296782 PMC8979958

[124] Beier UH Wang L Bhatti TR Liu Y Han R Ge G Sirtuin-1 targeting promotes Foxp3+ T-regulatory cell function and prolongs allograft survival. Mol Cell Biol. (2011) 31(5):1022–9. 10.1128/MCB.01206-10 21199917 PMC3067815

[125] Malaguarnera L. Influence of resveratrol on the immune response. Nutrients. (2019) 11(5). 10.3390/nu11050946 PMC6566902 31035454

[126] Prietl B Treiber G Pieber TR Amrein K. Vitamin D and immune function. Nutrients. (2013) 5(7):2502–21. 10.3390/nu5072502 23857223 PMC3738984

[127] Buyukdemirci S Oguz EG Cimen SG Sahin H Cimen S Ayli MD. Vitamin D deficiency may predispose patients to increased risk of kidney transplant rejection. World J Transplant. (2022) 12(9):299–309. 10.5500/wjt.v12.i9.299 36187881 PMC9516489

[128] Obi Y Hamano T Ichimaru N Tomida K Matsui I Fujii N Vitamin D deficiency predicts decline in kidney allograft function: a prospective cohort study. J Clin Endocrinol Metab. (2014) 99(2):527–35. 10.1210/jc.2013-2421 24285688

[129] Bienaimé F Girard D Anglicheau D Canaud G Souberbielle JC Kreis H Vitamin D status and outcomes after renal transplantation. J Am Soc Nephrol. (2013) 24(5):831–41. 10.1681/ASN.2012060614 23539758 PMC3636791

[130] Bai YJ Li YM Hu SM Zou YG An YF Wang LL Vitamin D supplementation reduced blood inflammatory cytokines expression and improved graft function in kidney transplant recipients. Front Immunol. (2023) 14:1152295. 10.3389/fimmu.2023.1152295 37483634 PMC10358325

